# Impulse oscillometry with quantitative computed tomography provides additional clinical information beyond spirometry in chronic airflow obstruction: a pilot study

**DOI:** 10.3389/fphys.2026.1851011

**Published:** 2026-06-15

**Authors:** Gyuri Kim, Hyonsoo Joo, Hyeon-Kyoung Koo, Ji-Yong Moon, Kwang Ha Yoo, Youlim Kim, Sanghun Choi

**Affiliations:** 1 School of Mechanical Engineering & Research Institute of Engineering Design & Technology (IEDT), Kyungpook National University, Daegu, Republic of Korea; 2 Division of Pulmonary and Critical Care Medicine, Department of Internal Medicine, Uijeongbu St. Mary’s Hospital, College of Medicine, The Catholic University of Korea, Seoul, Republic of Korea; 3 Division of Pulmonary and Critical Care Medicine, Department of Internal Medicine, Inje University Ilsan Paik Hospital, Inje University College of Medicine, Goyang, Republic of Korea; 4 Division of Pulmonary, Allergy, and Critical Care Medicine, Department of Internal Medicine, Konkuk University Medical Center, Konkuk University School of Medicine, Seoul, Republic of Korea

**Keywords:** impulse oscillometry, pulmonary function test, pulmonary reactance, quantitative computed tomography, spirometry

## Abstract

**Background:**

Conventional spirometric indices such as forced expiratory volume in 1 second (FEV_1_), forced vital capacity (FVC), and its ratio, FEV_1_/FVC, are commonly used to assess functional alterations in lungs. Quantitative computed tomography (qCT) and impulse oscillometry (IOS) provide additional insights on the traditional metrics. This study aims to investigate the interrelationship among spirometric, oscillometric, and qCT measures in subjects with varying pulmonary function.

**Methods:**

Sixty-four subjects with variable pulmonary functions were recruited and stratified into four sub-groups (G1-G4) based on spirometric severity defined by FVC and FEV_1_/FVC. The qCTs at full inspiration and normal expiration enable image registration-based metrics, including the determinant of the Jacobian (Jacobian), the anisotropic deformation index (ADI), and functional small airway disease (fSAD%). We also obtained IOS metrics to measure reactance (X5 through X20), resonant frequency (f_res_) and reactance area (A_X_) for respective subjects.

**Results:**

IOS-based reactances at 10 and 15 Hz (X10 and X15) were significantly more negative in groups with reduced FVC (G2 and 4) than in those with preserved FVC (G1 and 3), whereas resistances did not differ significantly between the sub-groups. The Jacobian and ADI were predominantly decreased in Groups 2 and 4 with FVC<80%. fSAD% was predominantly increased in Groups 3 and 4 with FEV_1_/FVC<70%. In particular, the X10 was most significantly correlated with Jacobian in the left and right lower lobes.

**Conclusion:**

The IOS-based reactances with low frequency were significantly associated with spirometry-pulmonary function and CT-based parenchymal lung function metrics. This finding suggests that a combined approach using IOS and CT can provide a better understanding of parenchymal alterations than spirometry alone.

## Introduction

Diagnosis of chronic obstructive pulmonary disease (COPD) requires spirometry to demonstrate persistent airflow limitation (Global Initiative for Chronic Obstructive Lung Disease, [[NoYear]]). However, spirometry requires active patient cooperation and forceful expiratory efforts, making it challenging to administer to individuals with cognitive impairment, limited physical ability, or poor coordination (Bickel et al., 2014; Kaminsky et al., 2022). Moreover, spirometric indices may not fully capture the regional and mechanistic heterogeneity of airway and parenchymal abnormalities. To address these limitations, impulse oscillometry (IOS), a non-invasive and simplified variant of the forced oscillation technique offers several advantages. IOS assesses respiratory impedance by superimposing sound waves onto normal tidal breathing and measuring the resultant changes in airway pressure and flow using fast Fourier transform algorithms (Desai and Joshi, 2019). Because IOS can detect airway obstruction during quiet breathing without requiring forceful maneuvers, it is particularly suitable for children, older adults, and patients unable to perform conventional Pulmonary Function Tests(PFT) reliably (Hayes et al., 1979). Recent advances in quantitative computed tomography (qCT) image processing have enabled the extraction of region-specific structural and functional metrics in pulmonary disease research (Kim et al., 2020). qCT facilitates the precise quantification of lesion distribution, airway morphology, and parenchymal abnormalities. In COPD, qCT has demonstrated its ability of quantifying emphysema and functional small airway disease (fSAD%). In addition, image registration allows to quantify regional lung deformation, such as the Jacobian determinant and anisotropic deformation index (ADI), indicating local expansion and geometric distortion of lung parenchyma (Koo et al., 2023).

Previous studies have explored the relationships between spirometry-defined airflow limitation and structural and functional assessments using either qCT or IOS in COPD. Airway morphometry on inspiratory CT, including peripheral-to-central airway lumen area ratios and percentage wall area, correlates significantly with COPD severity across GOLD stages (Sasaki et al., 2014). Parametric response mapping (PRM)-based analyses have shown that CT-defined functional small airway disease is significantly associated with subsequent declines in spirometric indices over longitudinal follow-up (Kim et al., 2025). Beyond these density-based measures, registration-based biomechanical metrics such as the Jacobian determinant and anisotropic deformation have also demonstrated strong correlations with FEV_1_ and improved concordance between spirometric assessment and CT-defined emphysema burden (Bhatt et al., 2016a). Concurrently, multiple clinical studies support the relevance of IOS to conventional spirometry. IOS resistance and reactance indices have shown consistent relationships with FEV_1_, FEV_1_/FVC, and COPD severity across GOLD stages, supported by multicenter cohorts and meta-analyses. Notably, reactance-related measures demonstrated stronger associations with spirometric impairment in patients with more severe airflow limitation (Wei et al., 2017; Gao et al., 2024; Peng et al., 2025). Collectively, these findings indicate that spirometry relates meaningfully to both IOS-derived respiratory mechanics and CT-derived imaging phenotypes. However, it remains unclear whether combined IOS–qCT profiles map onto distinct spirometric patterns, as most studies have evaluated these modalities within separate analytic frameworks, particularly when reduced lung volume and airflow obstruction coexist.

Therefore, the aim of this study is to investigate whether spirometric phenotypes defined by combined patterns of FVC, FEV_1_, and FEV_1_/FVC exhibit differential functional and structural characteristics on IOS and qCT. Participants were categorized into four spirometry-based groups, and we compared group-specific variations in IOS impedance indices and qCT-derived functional metrics, including deformation-based measures and PRM-derived fSAD%. We hypothesized that an integrated IOS–qCT assessment would better capture physiological heterogeneity beyond conventional spirometry alone.

## Methods

### Study subjects

**Figure 1:**
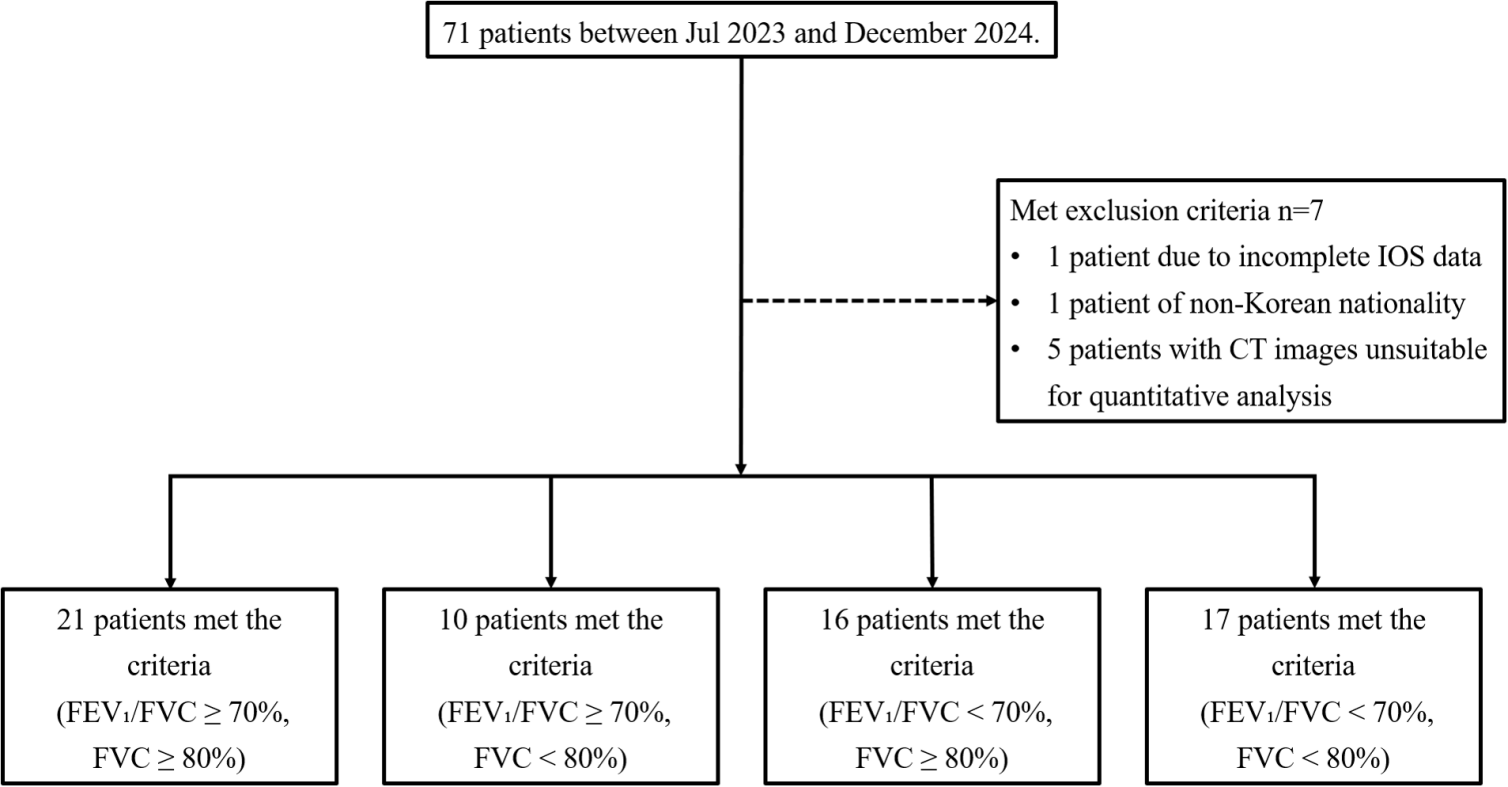
Flow diagram of study participant inclusion and exclusion.

Participants were retrospectively identified from Konkuk University Hospital. Eligible participants were adults with spirometry and IOS measurements, and with paired inspiratory and expiratory chest CT scans suitable for quantitative analysis (**Figure 1**).

Spirometry was performed to obtain FEV_1_, FVC, and the FEV_1_/FVC ratio. Airflow obstruction was defined as an FEV_1_/FVC ratio < 0.70. To capture lung volume-related heterogeneity, reduced lung volume was defined as FVC < 80% predicted. Participants were categorized into four spirometry-based phenotypes: (1) preserved FEV_1_/FVC with preserved FVC, (2) preserved FEV_1_/FVC with reduced FVC, (3) airflow obstruction with preserved FVC, and (4) airflow obstruction with reduced FVC. This approach was inspired by earlier research showing that FVC provides additional information on lung structural and functional changes beyond airflow-based indices and reflects volume-related physiological changes (An et al., 2022). PFT were performed using standard equipment, in accordance with the American Thoracic Society/European Respiratory Society (ATS/ERS) guidelines (Graham et al., 2019), with the percentage of pulmonary function predicted based on normal spirometry values adjusted for the Korean population. The study protocol was approved by the Institutional Review Board of Konkuk University Hospital (IRB No. KUMC 2025-07-010) and conducted in accordance with the Declaration of Helsinki. The requirement for informed consent was waived due to the retrospective study design.

### CT acquisition and image processing

All chest CT scans were acquired using multidetector CT scanners; scanner models and acquisition parameters are summarized in **Table 1**. Paired inspiratory and expiratory scans were reconstructed with a slice thickness of 1.0 or 1.25 mm, depending on the scanner and reconstruction protocol (**Table 1**).

**Table 1:** Scanner models and scanning protocols used for CT imaging.

Scanners and scanning protocol					
Institution	Konkuk University Medical Center				
Scanner model	Philips, Ingenuity CT	Siemens, Somatom X.ceed	Siemens, Somatom Force	GE, Optima CT660	GE, Revolution Apex
Scan type	Helical	Helical	Helical	Helical	Helical
Rotation time (s)	0.4 s	0.25 s	0.5 s	0.5 s	0.28 s
Detector configuration	64*0.625 mm	128*0.6 mm	128*0.6 mm	64*0.625 mm	256*0.625mm
Pitch	0.797	1.2	1.0	0.98	1.5
Peak kilovoltage (kVp)	120 kVp	120 kVp	120 kVp	120 kVp	100 kVp
mAs	100–162 mAs (AEC mode)	104 and 101 mAs	60 mAs	3 mAs	35–85 mAs (AEC mode)
Dose modulation	3D Modulation	Care Dose4D and Care kV	Care Dose4D and Care kV	Smart mA	Smart mA and Smart kV
Reconstruction algorithm	C and YC	Br40f\3 and Br60f\3	Br40d\3 and Br59d\3	Bone	Lung and Standard
Thickness (mm)	1.0 mm	1.0 mm	1.0 mm	1.25 mm	1.25 mm
Iterative reconstruction	iDose	ADMIRE	ADMIRE	ASiR	ASiR and TrueFidelity

**Figure 2:**
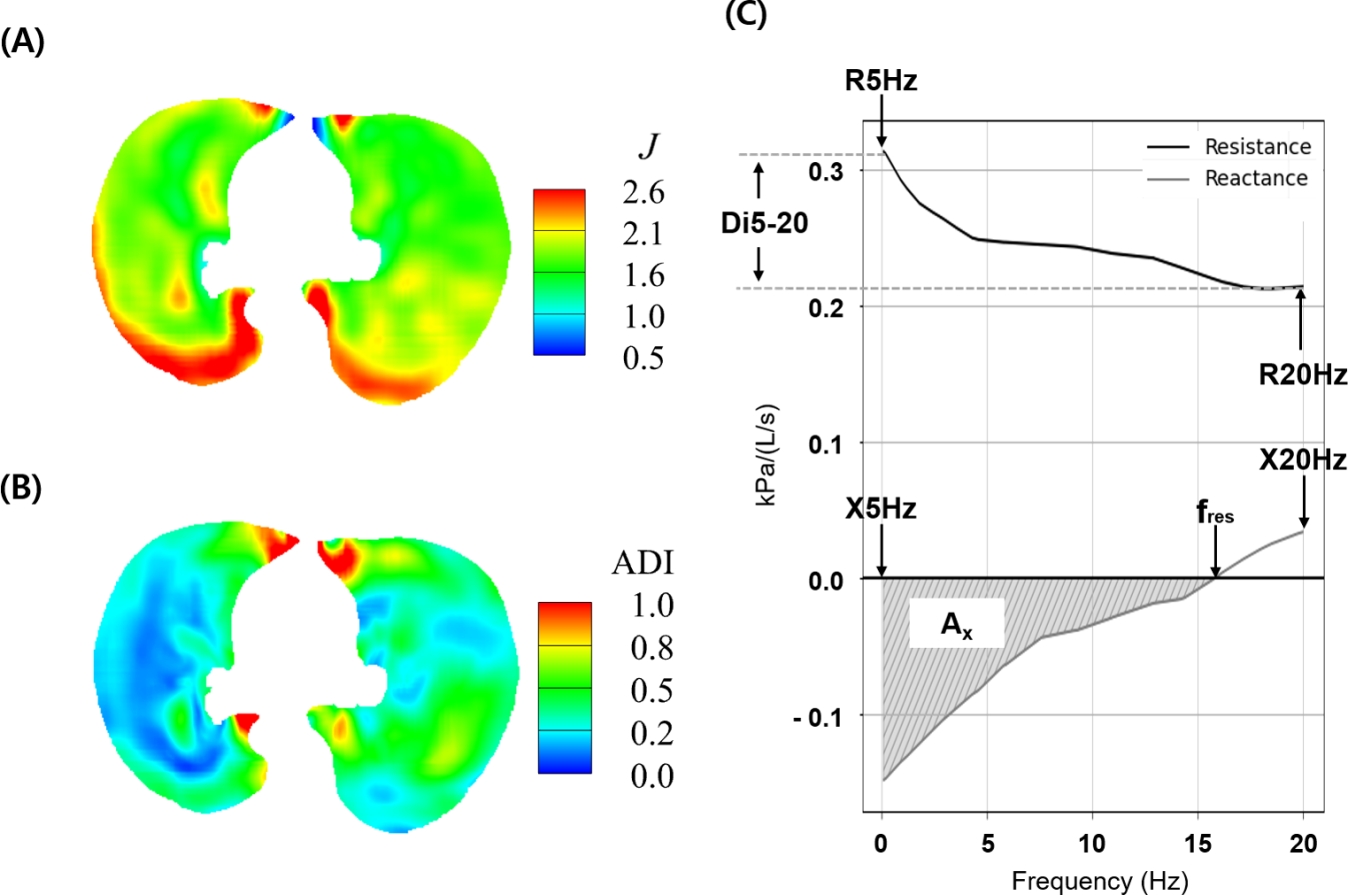
Integrated Overview of qCT-Derived and IOS-Derived Variables. **(A)** Voxel-wise distribution of Jacobian values indicating regional lung deformation. **(B)** Voxel-wise distribution of anisotropic deformation index (ADI) reflecting distal airspace geometry. **(C)** Schematic representation of impulse oscillometry system (IOS)-derived variables.

A mass-preserving image registration was applied to align expiratory CT images with their inspiratory counterparts to enable assessment of regional parenchymal deformation and ventilation abnormalities (Yin et al., 2009). Local volume changes were quantified using the Jacobian determinant, where values >1 indicated regional expansion, values <1 indicated compression, and a value of 1 indicated no volume change (**Figure 2A**) (Choi et al., 2013). This allowed direct evaluation of lung inflation and deflation across regions. The anisotropic deformation index (ADI) was computed to quantify directional heterogeneity in tissue deformation (**Figure 2B**) (Choi et al., 2013). A high ADI indicated directional distortion, while a low ADI reflected isotropic expansion. Together, the Jacobian and ADI provided a comprehensive assessment of both the magnitude and geometric distribution of regional lung deformation. In addition to deformation-based metrics, functional small airway disease (fSAD%) and emphysema were quantified using the PRM technique (Galbán et al., 2012). Co-registered inspiratory and expiratory CT scans were used to classify individual voxels based on their Hounsfield unit (HU) attenuation values.

Using the PRM method, voxels were classified as emphysematous if their attenuation values were below -950 HU on inspiratory CT and below -856 HU on expiratory CT. fSAD% was defined as the percentage of voxels with attenuation values greater than -950 HU on inspiratory CT and below -856 HU on expiratory CT, representing regions of air trapping in the absence of emphysema.

### IOS-based variables

IOS was performed using a Masterscreen IOS system (Master Lab-IOS, version 3.20, Germany) according to ERS recommendations (Bickel et al., 2014). Long-acting bronchodilators were withheld for 12 h and short-acting bronchodilators for 4 h before testing. Measurements were acquired during tidal breathing for 20–30 s over 5–35 Hz. Three technically acceptable tests meeting the reproducibility criteria were obtained. Respiratory impedance (Z) was partitioned into resistance (R) and reactance (X). We analyzed R5, R10, R15, and R20; X5, X10, X15, and X20; the resonant frequency (f_res_); the area of negative reactance (A_x_; 5 Hz to f_res_); and the frequency dependence of resistance (Di5–20) (Desai and Joshi, 2019). Reactance indices (X, A_x_, f_res_) were interpreted as reflecting the combined elastic and inertial properties of the respiratory system. A_x_ was defined as the area under the reactance curve from 5 Hz to f_res_ (**Figure 2C**) (Bickel et al., 2014).

### Statistical analysis

Homogeneity of variance across groups was assessed using Levene’s test implemented in the “scipy.stats” package. To account for unequal variances and sample sizes, Welch’s analysis of variance was performed using the “pingouin” and “statsmodels” libraries. *Post hoc* comparisons were conducted using the Games–Howell test in “pingouin”. Given the non-normal distribution of several variables, Spearman’s rank correlation coefficient was used to examine associations between CT-derived parameters and IOS measures. In addition, multivariable linear regression analyses were performed to evaluate whether the associations between qCT-derived metrics and IOS parameters remained significant after adjustment for age, sex, and height. To control for type I errors from multiple comparisons, false discovery rate (FDR) correction was applied using the Benjamini–Hochberg procedure implemented in the “statsmodels” library. Correlation matrices were generated and visualized using the “pandas,” “seaborn,” and “matplotlib” libraries. All statistical analyses were conducted using Python version 3.12.3.

## Results

### Study population

A total of 71 participants were screened (**Figure 1**). Seven were excluded due to incomplete IOS data (n=1), non-Korean nationality (n=1), or CT images unsuitable for quantitative analysis (n=5), leaving 64 participants for the final analysis. Baseline demographic and spirometric characteristics by group are summarized in **Table 2**. As expected, spirometric indices differed significantly across groups, reflecting the study definitions (all P<0.001). Age also differed across groups (P<0.01), with a higher mean age in the airflow obstruction groups. The prevalence of physician-reported emphysema and prior tuberculosis differed by group (P = 0.006 and P = 0.049, respectively).

**Table 2:** Demographic and pulmonary function test results.

Characteristic	Group 1	Group 2	Group 3	Group 4	p-value
	(N = 21)	(N = 10)	(N = 16)	(N = 14)	
Demography					
Sex, no. (% women)	3 (14.3)	4 (40.0)	3 (18.8)	5 (29.4)	0.39
Smoking participants, (% current and former smokers)	17 (81.0)	6 (60.0)	13 (81.2)	8 (47.1)	0.08
Age, years	57.8 (9.7)	61.1 (13.6)	67.3 (11.2)	69.4 (8.2)	<0.01
Height, cm	167.2 (12.2)	163.5 (13.4)	165.0 (9.9)	162.2 (7.5)	0.49
BMI, kg/m^2^	24.4 (3.1)	27.4 (5.0)	22.8 (3.6)	24.0 (3.9)	0.12
Spirometry					
FEV_1_, %predicted	93.0 (9.7)	73.0 (8.8)	78.3 (18.6)	57.7 (14.7)	<0.001
FVC, %predicted	91.0 (7.7)	71.1 (5.2)	94.8 (14.0)	67.3 (8.5)	<0.001
FEV_1_/FVC, %predicted	77.5 (5.6)	75.3 (6.4)	59.3 (9.0)	60.1 (10.6)	<0.001
Comorbidities					
emphysema (n, %)	3 (14.3)	2 (20.0)	10 (62.5)	3 (17.6)	0.006
tuberculosis (n, %)	3 (14.3)	3 (30.0)	0 (0.0)	6 (35.3)	0.049
bronchiectasis (n, %)	2 (9.5)	2 (20.0)	5 (31.2)	5 (29.4)	0.35

Values are presented as mean (standard deviation). BMI, body mass index; FEV_1_, forced expiratory volume in 1 second; FVC, forced vital capacity.

### Comparison of IOS variables among groups

**Figure 3:**
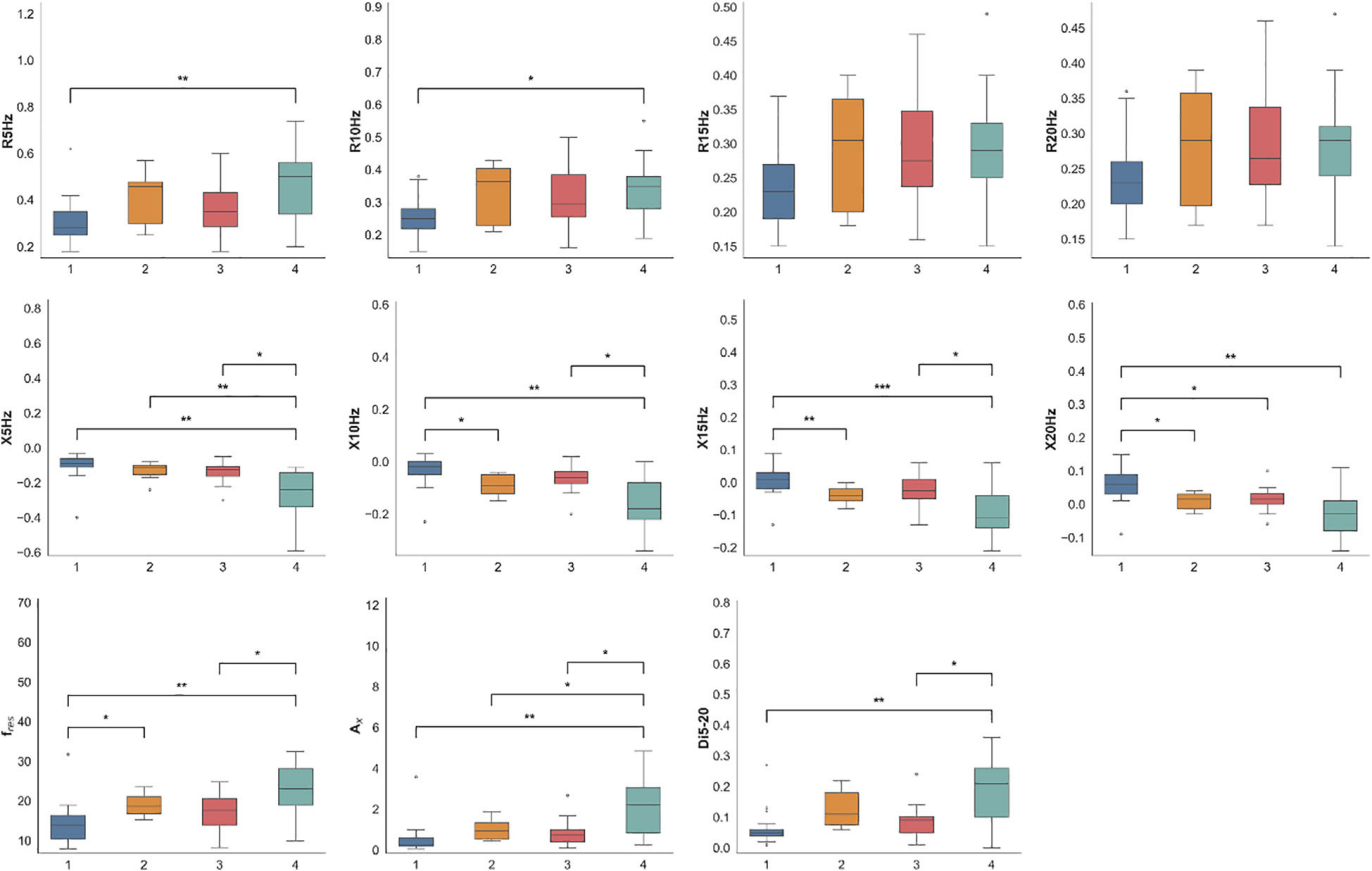
Comparison of post-bronchodilator IOS variables across groups stratified by airflow obstruction and lung volume status. *(*p* < 0.05), **(*p* < 0.01), ***(*p* < 0.001).

Reactance-related IOS parameters showed distinct differences between groups based on lung volume status and the existence of airflow obstruction (**Figure 3**). With increasing functional impairment, reactance values (X5 through X20) became progressively more negative, with the most pronounced separation observed at lower frequencies. Within each airflow category, reduced FVC was associated with more negative intermediate-frequency reactance. Specifically, groups with reduced FVC showed significantly more negative X10 and X15 compared with their preserved-FVC counterparts, regardless of airflow obstruction status. Reactance-based summary indices showed concordant patterns: f_res_ increased in reduced FVC groups and peaked in those with both airflow obstruction and reduced FVC, while A_x_ demonstrated a stepwise increase across phenotypes.

### Comparison of qCT variables among groups

**Figure 4:**
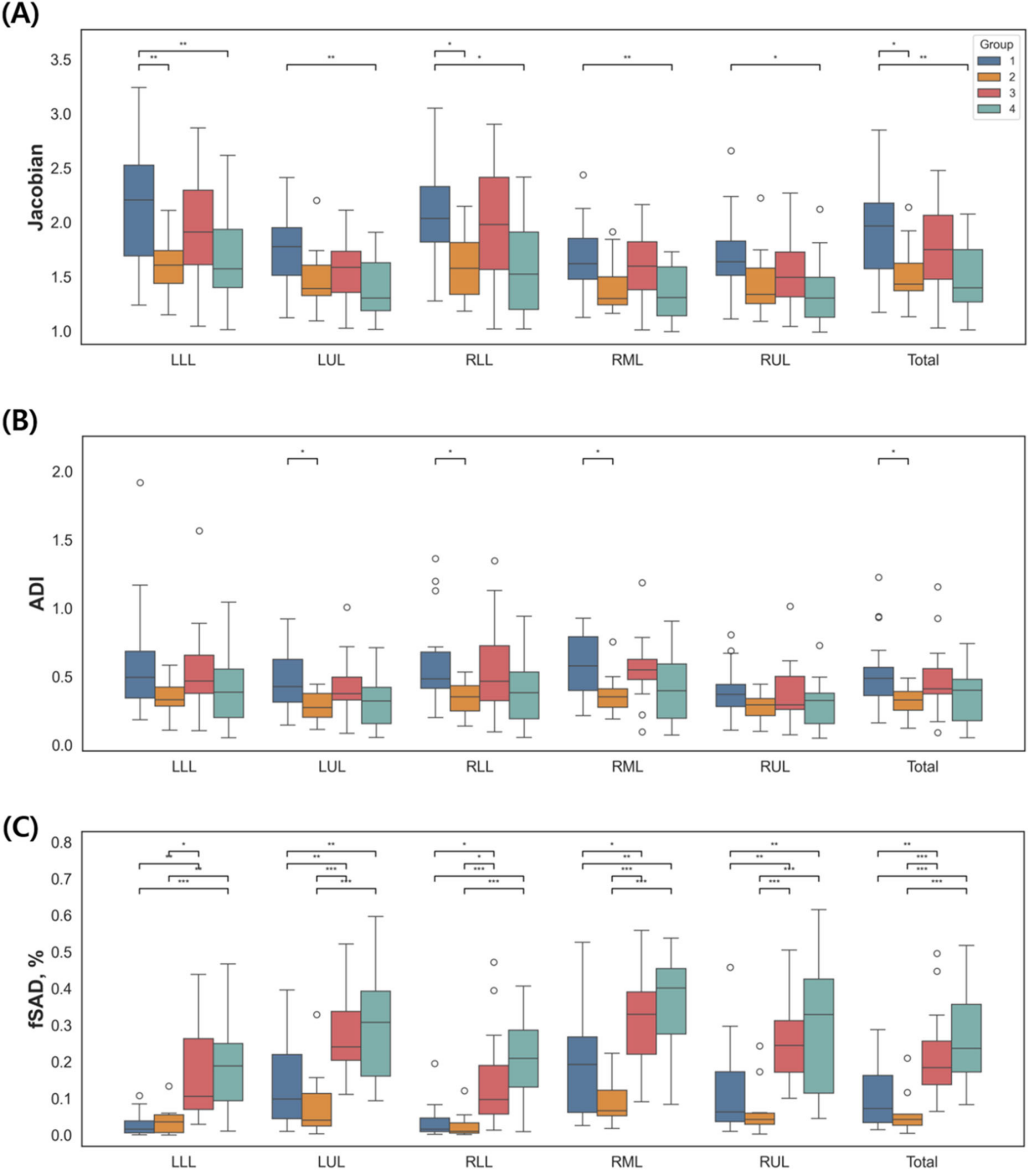
Comparison of qCT-derived functional indices across groups. *(*p* < 0.05); **(*p* < 0.01); ***(*p* < 0.001). **(A)** Jacobian; **(B)** Anisotropic deformation index (ADI); **(C)** Functional small airway disease percentage (fSAD%).

**Figure 5:**
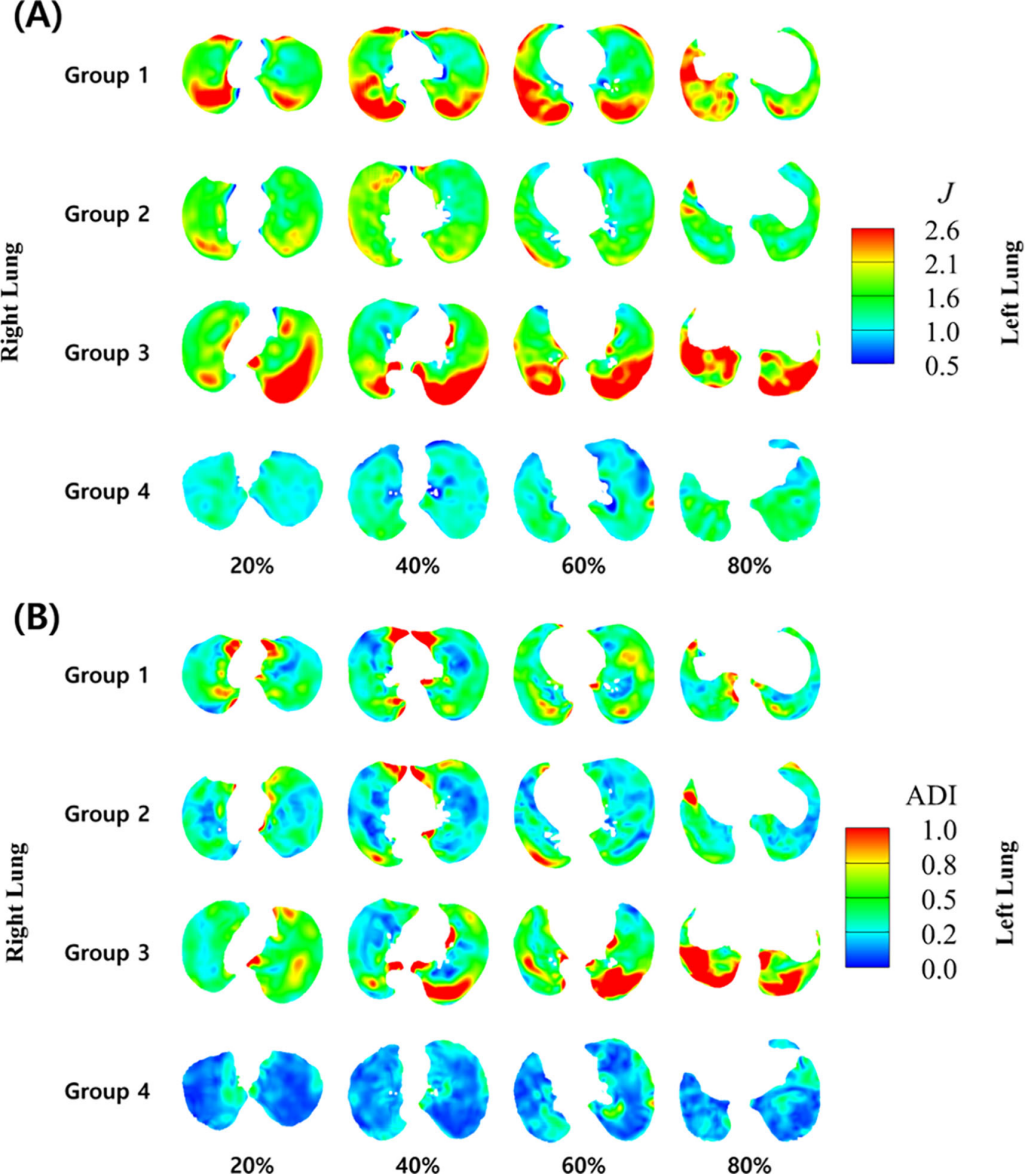
Spatial distributions for a representative subject are presented at 20%, 40%, 60%, 80% from the apical to basal regions. **(A)** Jacobian; **(B)** ADI.

qCT-derived functional indices demonstrated group-dependent differences in regional lung deformation and small airway disease (**Figure 4**). Jacobian values were generally lower in reduced-lung-volume groups at both whole-lung and regional levels, with significant differences observed primarily compared with preserved FEV_1_/FVC with preserved FVC group. ADI also showed significant group effects at the whole-lung level and across most lobes. fSAD% showed clear separation across groups all lobes and at the whole-lung level, with the most pronounced differences observed in groups with airflow obstruction. Representative voxel-wise spatial distributions for Jacobian and ADI are shown in **Figure 5**, where groups with preserved FVC exhibited a higher frequency of elevated deformation values compared with reduced FVC groups.

### Correlation between qCT variables and IOS variables

**Figure 6:**
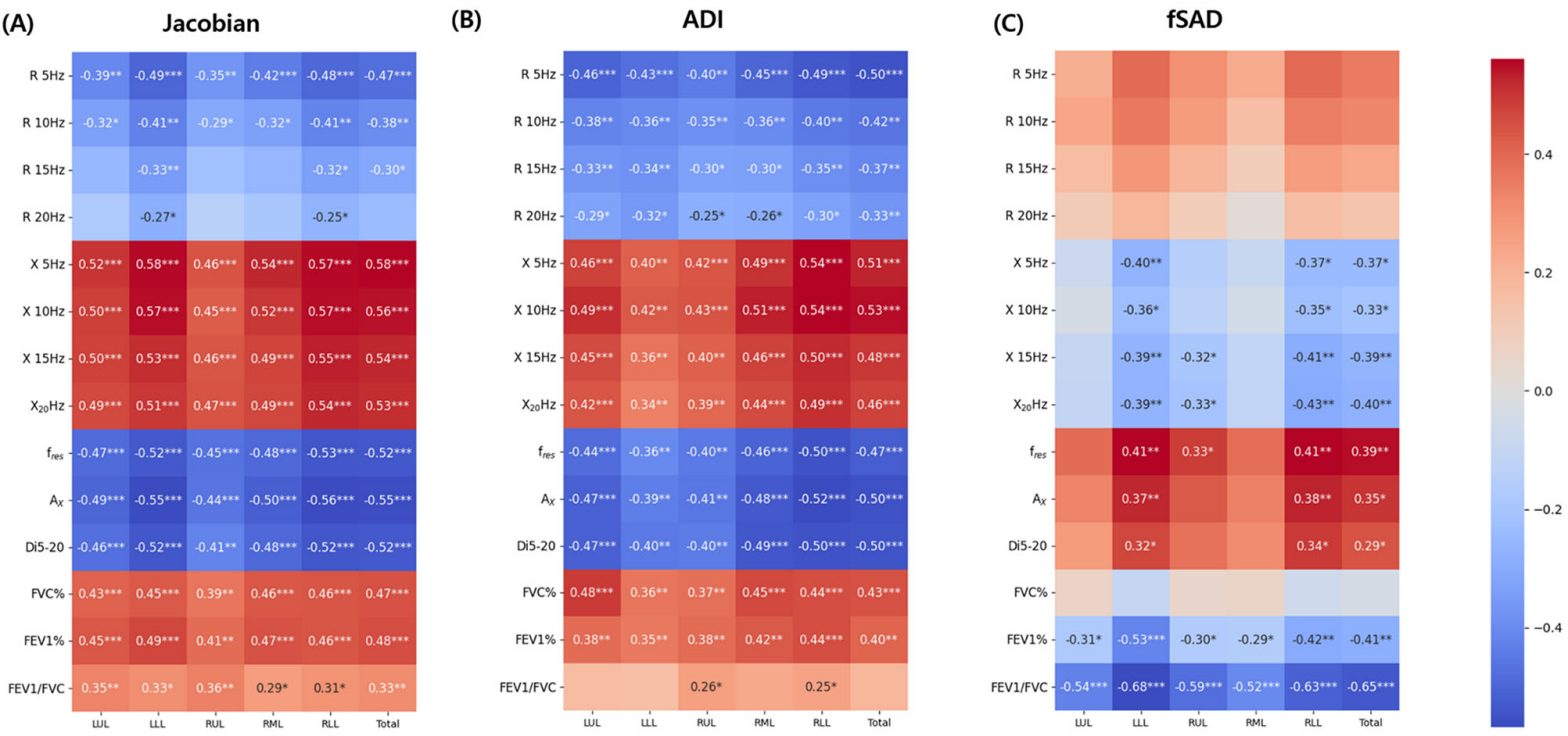
Spearman correlation heatmaps between IOS variables and qCT-derived indices. Correlation strength is indicated by values approaching 1 (positive), –1 (negative), or 0 (no correlation). *(*p* < 0.05), **(*p* < 0.01), ***(*p* < 0.001). **(A)** Jacobian; **(B)** ADI; **(C)** fSAD%.

The associations between IOS parameters and qCT-derived functional metrics after FDR correction are presented in **Figure 6**. Functional deformation metrics demonstrated strong correlations with IOS indices. Lower Jacobian values were significantly correlated with smaller reactance (X5 through X20), as well as increased f_res_ and A_x_ values (**Figure 6A**). These correlations were consistently observed across all lobes, but were more prominent in the lower lobes. Jacobian also shows positive associations with spirometric indices such as FVC% and FEV_1_%. Similar trends were observed for ADI, where lower values correlated with reduced reactance, FVC%, and elevated f_res_ and A_x_ (**Figure 6B**). Increased fSAD% was significantly associated with reduced reactance especially in the left lower lobe (LLL), and right lower lobe (RLL), consistent with Jacobian and ADI. However, there was no significant correlation between fSAD% and FVC%, whereas a strong negative correlation was observed between fSAD% and FEV_1_/FVC (**Figure 6C**). Detailed numerical results, including Spearman’s rho, nominal p-values, and FDR-adjusted p-values, are provided in **Supplementary Tables 1**–**3**. Multivariable linear regression analyses adjusting for age, sex, and height were performed with IOS parameters as dependent variables and qCT-derived metrics as the main independent variables, and the results are provided in **Supplementary Tables 4**–**6**.

## Discussion

In this study, we characterized mechanical and imaging phenotypes across four spirometry-defined groups stratified by airflow obstruction (FEV_1_/FVC < 0.70) and reduced FVC (FVC < 80% predicted), using an integrated assessment of IOS and qCT. We found that IOS reactance-related indices (X5 through X20, A_X_, and f_res_) were consistently more abnormal in groups with reduced FVC, even in the absence of airflow obstruction, indicating that reduced lung volume exerts a dominant influence on oscillometric reactance at intermediate frequencies. In addition, qCT registration-derived deformation metrics (Jacobian and ADI) were significantly lower in the reduced-FVC groups and showed strong associations with reactance parameters, supporting a mechanistic link between impaired regional expansion and abnormal elastic behavior measured during tidal breathing (Sahin et al., 2023). Finally, PRM-derived fSAD% tracked airflow obstruction (FEV_1_/FVC) more closely than reduced FVC, suggesting that volume reduction and obstruction represent partially independent physiological dimensions that map onto distinct imaging and mechanical signatures. Overall, these results show that IOS and qCT provide complementary and mechanistically interpretable information beyond spirometry alone, linking reactance abnormalities to regional deformation, whereas PRM-fSAD aligns more closely with airflow obstruction.

Interpretation of the IOS findings is clinically relevant because reactance captures volume-dependent elastic behavior during quiet tidal breathing. Although X5 is the most widely reported reactance parameter, respiratory reactance is inherently frequency-dependent. At lower frequencies, reactance is more strongly influenced by the elastic properties of the lung and chest wall, whereas with increasing frequency, the contribution of inertance increases and reactance becomes less negative (King et al., 2020). Across spirometric phenotypes, intermediate-frequency reactance (X10 and X15) was consistently more negative in participants with reduced FVC regardless of airflow obstruction. This pattern suggests that reduced lung volume can dominate the influence on the oscillometric reactance signature, potentially reflecting increased elastic loading or altered regional lung expansion (Takeichi et al., 2019).

The qCT-derived indices help explain why reduced FVC was associated with reactance abnormalities. The Jacobian summarizes local volumetric change from expiration to inspiration, whereas ADI captures directional heterogeneity of tissue deformation (Yin et al., 2009; Choi et al., 2013). Lower Jacobian and ADI values in the reduced-FVC groups indicate diminished regional expandability and more constrained deformation, consistent with reduced effective compliance (Takeichi et al., 2019). Importantly, correlations between parenchymal functional metrics and reactance-related IOS indices were observed across lobes and appeared most pronounced in the lower lobes. Basal lung regions are sensitive to gravitational effects and regional differences in ventilation and tissue mechanics; therefore, lower-lobe deformation impairment may disproportionately contribute to volume-dependent reactance abnormalities detected during tidal breathing (Kaminsky et al., 2022). Notably, Jacobian and ADI quantify different aspects of deformation. Their associations with reactance are similar in direction and magnitude, suggesting that both reduced expansion and increased deformation heterogeneity contribute to the oscillometric signature of reduced FVC in this cohort (Fortis et al., 2020).

In contrast to deformation-based measures (Bodduluri et al., 2017), PRM-derived fSAD% demonstrated a different relationship to spirometry. PRM-fSAD identifies regions with relatively preserved inspiratory attenuation but increased expiratory air trapping, reflecting functional small airway abnormalities central to COPD pathophysiology and disease progression (Galbán et al., 2012; Bhatt et al., 2016b; Vasilescu et al., 2019). In our study, fSAD% was significantly associated with FEV_1_/FVC but not with FVC, supporting a dissociation between volume-related mechanical constraints and obstruction-related small airway dysfunction. Although the association between f_res_ and small airway involvement has been previously described (Su et al., 2018), the present study extends this concept by integrating IOS parameters with spirometric indices and qCT-derived imaging variables within the same cohort. It also underscores the importance of distinguishing airway obstruction from lung volume reduction when interpreting imaging biomarkers: reduced FVC does not necessarily imply more extensive fSAD, and conversely, substantial fSAD can exist across a range of FVC values depending on the balance of air trapping, hyperinflation, and expiratory flow limitation (O'Donnell, 2006). Our results suggest a mechanistic distinction in which PRM-fSAD tracks airway-level expiratory trapping linked to obstruction, while Jacobian or ADI captures deformation and volume-related mechanics more closely reflected in reactance, clarifying that reduced FVC and airway obstruction do not reflect a single severity continuum but rather represent complementary physiologic axes corresponding to distinct functional domains (Schroeder et al., 2013).

These findings have several important implications for phenotyping and clinical applications. First, IOS may provide a sensitive, effort-independent index for measuring volume-dependent compliance abnormalities during resting breathing, particularly in patients with reduced FVC, where forced expiratory effort is variable and spirometry alone cannot distinguish between restrictive and obstructive patterns of reduced vital capacity. Second, when CT is already clinically available, qCT may allow a regional mechanistic interpretation of IOS abnormalities by spatially linking volume-dependent reactance changes to CT-derived deformation impairment, such as reduced Jacobian and ADI, and by distinguishing these deformation-related signals from obstruction-related PRM-fSAD. This integrated approach is particularly useful when spirometry results are inconsistent or symptoms are more severe than reported by conventional spirometry, providing orthogonal information on deformation, ventilatory inhomogeneity, and small airway dysfunction. Third, our results support that a phenotypic classification based on two axes, airway obstruction and lung volume status, can better capture clinically meaningful heterogeneity than a classification based solely on obstruction, and may provide a useful framework for future studies investigating prognosis or treatment response.

IOS complements spirometry by measuring respiratory impedance during quiet tidal breathing and provides effort-independent information on the mechanical properties of the respiratory system, including the airways, lung tissue, and chest wall (Bickel et al., 2014; King et al., 2020). However, IOS parameters remain global mechanical signals and do not provide direct anatomical localization. Therefore, the added value of qCT lies in its ability to provide regional imaging-based information that can help interpret global IOS abnormalities (Yin et al., 2009; Galbán et al., 2012; Choi et al., 2013). This study has the following strengths. We applied a prespecified spirometry-based stratification that explicitly incorporates lung volume reduction alongside airflow obstruction, enabling evaluation of two complementary physiological dimensions that are not fully disentangled by spirometry alone. We also integrated complementary modalities, oscillometric impedance and both density-based (PRM-fSAD) and registration-based (Jacobian and ADI) qCT metrics, allowing triangulation of functional findings with plausible regional structural correlates. Finally, we performed lobe-wise analyses, which helped localize structure–function relationships and highlighted potential regional predominance of deformation–reactance coupling.

This study has several limitations. First, this was a retrospective and cross-sectional study based on clinical and imaging data obtained during routine clinical practice. Therefore, no *a priori* sample size calculation was performed, and causal or temporal relationships among airflow obstruction, reduced lung volume, IOS abnormalities, and qCT-derived imaging metrics cannot be determined. Second, Heterogeneous mechanisms, such as submaximal inspiration, altered respiratory mechanics, or true parenchymal restriction, may result in reduced lung volume; however, these mechanisms cannot be fully distinguished within the current study design. Third, the study population was drawn from a single cohort, and additional validation is necessary to determine whether the results can be applied to other populations or clinical settings. Fourth, respiratory phase-specific IOS parameters, including inspiratory and expiratory X5, AX, and R5, were not available in the current retrospective dataset. Future prospective studies should incorporate phase-resolved IOS measurements to better evaluate the dynamic relationship between qCT-derived deformation metrics and oscillometric mechanics. Fifth, the relatively small sample size, particularly within each spirometry-defined subgroup, limits the generalizability of the findings. Therefore, the present results should be interpreted as exploratory and hypothesis-generating, and external validation in larger multicenter cohorts is required. Lastly, the lack of detailed clinical data, including symptom burden and medication history, limits the ability to contextualize the findings within a broader clinical framework. Future research should incorporate these variables to enable more precise phenotypic characterization.

In conclusion, spirometry-based stratification by airflow obstruction and reduced FVC revealed complementary abnormalities on IOS and qCT, reactance linking to reduced lung volume and deformation, and PRM-fSAD aligning with airflow obstruction, supporting an integrated approach to disentangle volume-dependent elastic loading from obstruction-driven small airway dysfunction. Future research should validate the longitudinal stability and clinical relevance of the IOS–qCT signatures identified here.

## Data Availability

The data analyzed in this study is subject to the following licenses/restrictions: The datasets analyzed in this study are not publicly available due to institutional and ethical restrictions related to patient confidentiality. Requests to access these datasets should be directed to Sanghun Choi, s-choi@knu.ac.kr.
