# Supplementary material for: Impulse oscillometry with quantitative computed tomography provides additional clinical information beyond spirometry in chronic airflow obstruction: a pilot study
Source: Front Physiol. 2026 Jun 15;17:1851011. doi: 10.3389/fphys.2026.1851011 (PMC13310701; doi:10.3389/fphys.2026.1851011)
Supplement: Supplementary file 1 [file Table1.docx]

*Supplementary*

Table S1. Spearman correlations between IOS variables and qCT-derived Jacobian.

|  |  | Spearman ρ | p-value | FDR-adjusted p-value |
| --- | --- | --- | --- | --- |
| R5 | LUL | -0.39 | <0.01 | <0.01 |
|  | LLL | -0.49 | <0.001 | <0.001 |
|  | RUL | -0.35 | <0.01 | <0.01 |
|  | RML | -0.42 | <0.001 | <0.001 |
|  | RLL | -0.48 | <0.001 | <0.001 |
|  | Total | -0.47 | <0.001 | <0.001 |
| R10 | LUL | -0.32 | <0.01 | 0.01 |
|  | LLL | -0.41 | <0.01 | <0.01 |
|  | RUL | -0.29 | 0.02 | 0.02 |
|  | RML | -0.32 | <0.01 | 0.01 |
|  | RLL | -0.41 | <0.01 | <0.01 |
|  | Total | -0.38 | <0.01 | <0.01 |
| R15 | LUL | -0.24 | 0.06 | 0.06 |
|  | LLL | -0.33 | <0.01 | <0.01 |
|  | RUL | -0.2 | 0.11 | 0.11 |
|  | RML | -0.24 | 0.06 | 0.06 |
|  | RLL | -0.32 | 0.01 | 0.01 |
|  | Total | -0.3 | 0.02 | 0.02 |
| R20 | LUL | -0.16 | 0.2 | 0.2 |
|  | LLL | -0.27 | 0.03 | 0.03 |
|  | RUL | -0.13 | 0.32 | 0.32 |
|  | RML | -0.18 | 0.15 | 0.16 |
|  | RLL | -0.25 | 0.04 | 0.05 |
|  | Total | -0.22 | 0.07 | 0.08 |
| X5 | LUL | 0.52 | <0.001 | <0.001 |
|  | LLL | 0.58 | <0.001 | <0.001 |
|  | RUL | 0.46 | <0.001 | <0.001 |
|  | RML | 0.54 | <0.001 | <0.001 |
|  | RLL | 0.57 | <0.001 | <0.001 |
|  | Total | 0.58 | <0.001 | <0.001 |
| X10 | LUL | 0.5 | <0.001 | <0.001 |
|  | LLL | 0.57 | <0.001 | <0.001 |
|  | RUL | 0.45 | <0.001 | <0.001 |
|  | RML | 0.52 | <0.001 | <0.001 |
|  | RLL | 0.57 | <0.001 | <0.001 |
|  | Total | 0.56 | <0.001 | <0.001 |
| X15 | LUL | 0.5 | <0.001 | <0.001 |
|  | LLL | 0.53 | <0.001 | <0.001 |
|  | RUL | 0.46 | <0.001 | <0.001 |
|  | RML | 0.49 | <0.001 | <0.001 |
|  | RLL | 0.55 | <0.001 | <0.001 |
|  | Total | 0.54 | <0.001 | <0.001 |
| X20 | LUL | 0.49 | <0.001 | <0.001 |
|  | LLL | 0.51 | <0.001 | <0.001 |
|  | RUL | 0.47 | <0.001 | <0.001 |
|  | RML | 0.49 | <0.001 | <0.001 |
|  | RLL | 0.54 | <0.001 | <0.001 |
|  | Total | 0.53 | <0.001 | <0.001 |
| f_res_ | LUL | -0.47 | <0.001 | <0.001 |
|  | LLL | -0.52 | <0.001 | <0.001 |
|  | RUL | -0.45 | <0.001 | <0.001 |
|  | RML | -0.48 | <0.001 | <0.001 |
|  | RLL | -0.53 | <0.001 | <0.001 |
|  | Total | -0.52 | <0.001 | <0.001 |
| A_x_ | LUL | -0.49 | <0.001 | <0.001 |
|  | LLL | -0.55 | <0.001 | <0.001 |
|  | RUL | -0.44 | <0.001 | <0.001 |
|  | RML | -0.5 | <0.001 | <0.001 |
|  | RLL | -0.56 | <0.001 | <0.001 |
|  | Total | -0.55 | <0.001 | <0.001 |
| Di5-20 | LUL | -0.46 | <0.001 | <0.001 |
|  | LLL | -0.52 | <0.001 | <0.001 |
|  | RUL | -0.41 | <0.001 | <0.01 |
|  | RML | -0.48 | <0.001 | <0.001 |
|  | RLL | -0.52 | <0.001 | <0.001 |
|  | Total | -0.52 | <0.001 | <0.001 |
| FVC% | LUL | 0.43 | <0.001 | <0.001 |
|  | LLL | 0.45 | <0.001 | <0.001 |
|  | RUL | 0.39 | <0.01 | <0.01 |
|  | RML | 0.46 | <0.001 | <0.001 |
|  | RLL | 0.46 | <0.001 | <0.001 |
|  | Total | 0.47 | <0.001 | <0.001 |
| FEV_1_% | LUL | 0.45 | <0.001 | <0.001 |
|  | LLL | 0.49 | <0.001 | <0.001 |
|  | RUL | 0.41 | <0.001 | <0.01 |
|  | RML | 0.47 | <0.001 | <0.001 |
|  | RLL | 0.46 | <0.001 | <0.001 |
|  | Total | 0.48 | <0.001 | <0.001 |
| FEV_1_/FVC | LUL | 0.35 | <0.01 | <0.01 |
|  | LLL | 0.33 | <0.01 | 0.01 |
|  | RUL | 0.36 | <0.01 | <0.01 |
|  | RML | 0.29 | 0.02 | 0.02 |
|  | RLL | 0.31 | 0.01 | 0.01 |
|  | Total | 0.33 | <0.01 | <0.01 |

Table S2. Spearman correlations between IOS variables and qCT-derived ADI.

|  |  | Spearman ρ | p-value | FDR-adjusted p-value |
| --- | --- | --- | --- | --- |
| R5 | LUL | -0.46 | <0.001 | <0.001 |
|  | LLL | -0.43 | <0.001 | <0.001 |
|  | RUL | -0.4 | <0.01 | <0.01 |
|  | RML | -0.45 | <0.001 | <0.001 |
|  | RLL | -0.49 | <0.001 | <0.001 |
|  | Total | -0.5 | <0.001 | <0.001 |
| R10 | LUL | -0.38 | <0.01 | <0.01 |
|  | LLL | -0.36 | <0.01 | <0.01 |
|  | RUL | -0.35 | <0.01 | <0.01 |
|  | RML | -0.36 | <0.01 | <0.01 |
|  | RLL | -0.4 | <0.001 | <0.01 |
|  | Total | -0.42 | <0.001 | <0.01 |
| R15 | LUL | -0.33 | <0.01 | <0.01 |
|  | LLL | -0.34 | <0.01 | <0.01 |
|  | RUL | -0.3 | 0.02 | 0.02 |
|  | RML | -0.3 | 0.02 | 0.02 |
|  | RLL | -0.35 | <0.01 | <0.01 |
|  | Total | -0.37 | <0.01 | <0.01 |
| R20 | LUL | -0.29 | 0.02 | 0.02 |
|  | LLL | -0.32 | 0.01 | 0.01 |
|  | RUL | -0.25 | 0.05 | 0.05 |
|  | RML | -0.26 | 0.04 | 0.04 |
|  | RLL | -0.3 | 0.02 | 0.02 |
|  | Total | -0.33 | <0.01 | <0.01 |
| X5 | LUL | 0.46 | <0.001 | <0.001 |
|  | LLL | 0.4 | <0.001 | <0.01 |
|  | RUL | 0.42 | <0.001 | <0.001 |
|  | RML | 0.49 | <0.001 | <0.001 |
|  | RLL | 0.54 | <0.001 | <0.001 |
|  | Total | 0.51 | <0.001 | <0.001 |
| X10 | LUL | 0.49 | <0.001 | <0.001 |
|  | LLL | 0.42 | <0.001 | <0.01 |
|  | RUL | 0.43 | <0.001 | <0.001 |
|  | RML | 0.51 | <0.001 | <0.001 |
|  | RLL | 0.54 | <0.001 | <0.001 |
|  | Total | 0.53 | <0.001 | <0.001 |
| X15 | LUL | 0.45 | <0.001 | <0.001 |
|  | LLL | 0.36 | <0.01 | <0.01 |
|  | RUL | 0.4 | <0.001 | <0.01 |
|  | RML | 0.46 | <0.001 | <0.001 |
|  | RLL | 0.5 | <0.001 | <0.001 |
|  | Total | 0.48 | <0.001 | <0.001 |
| X20 | LUL | 0.42 | <0.001 | <0.001 |
|  | LLL | 0.34 | <0.01 | <0.01 |
|  | RUL | 0.39 | <0.01 | <0.01 |
|  | RML | 0.44 | <0.001 | <0.001 |
|  | RLL | 0.49 | <0.001 | <0.001 |
|  | Total | 0.46 | <0.001 | <0.001 |
| f_res_ | LUL | -0.44 | <0.001 | <0.001 |
|  | LLL | -0.36 | <0.01 | <0.01 |
|  | RUL | -0.4 | <0.001 | <0.01 |
|  | RML | -0.46 | <0.001 | <0.001 |
|  | RLL | -0.5 | <0.001 | <0.001 |
|  | Total | -0.47 | <0.001 | <0.001 |
| A_x_ | LUL | -0.47 | <0.001 | <0.001 |
|  | LLL | -0.39 | <0.01 | <0.01 |
|  | RUL | -0.41 | <0.001 | <0.01 |
|  | RML | -0.48 | <0.001 | <0.001 |
|  | RLL | -0.52 | <0.001 | <0.001 |
|  | Total | -0.5 | <0.001 | <0.001 |
| Di5-20 | LUL | -0.47 | <0.001 | <0.001 |
|  | LLL | -0.4 | <0.001 | <0.01 |
|  | RUL | -0.4 | <0.01 | <0.01 |
|  | RML | -0.49 | <0.001 | <0.001 |
|  | RLL | -0.5 | <0.001 | <0.001 |
|  | Total | -0.5 | <0.001 | <0.001 |
| FVC% | LUL | 0.48 | <0.001 | <0.001 |
|  | LLL | 0.36 | <0.01 | <0.01 |
|  | RUL | 0.37 | <0.01 | <0.01 |
|  | RML | 0.45 | <0.001 | <0.001 |
|  | RLL | 0.44 | <0.001 | <0.001 |
|  | Total | 0.43 | <0.001 | <0.001 |
| FEV_1_% | LUL | 0.38 | <0.01 | <0.01 |
|  | LLL | 0.35 | <0.01 | <0.01 |
|  | RUL | 0.38 | <0.01 | <0.01 |
|  | RML | 0.42 | <0.001 | <0.01 |
|  | RLL | 0.44 | <0.001 | <0.001 |
|  | Total | 0.4 | <0.01 | <0.01 |
| FEV_1_/FVC | LUL | 0.17 | 0.19 | 0.19 |
|  | LLL | 0.16 | 0.2 | 0.2 |
|  | RUL | 0.26 | 0.04 | 0.04 |
|  | RML | 0.23 | 0.07 | 0.07 |
|  | RLL | 0.25 | 0.05 | 0.05 |
|  | Total | 0.19 | 0.13 | 0.14 |

Table S3. Spearman correlations between IOS variables and qCT-derived fSAD%.

|  |  | Spearman ρ | p-value | FDR-adjusted p-value |
| --- | --- | --- | --- | --- |
| R5 | LUL | 0.07 | 0.57 | 0.64 |
|  | LLL | 0.24 | 0.05 | 0.11 |
|  | RUL | 0.16 | 0.21 | 0.28 |
|  | RML | 0.08 | 0.52 | 0.6 |
|  | RLL | 0.25 | 0.05 | 0.1 |
|  | Total | 0.21 | 0.1 | 0.16 |
| R10 | LUL | 0.1 | 0.45 | 0.54 |
|  | LLL | 0.21 | 0.09 | 0.15 |
|  | RUL | 0.13 | 0.32 | 0.4 |
|  | RML | 0.02 | 0.86 | 0.88 |
|  | RLL | 0.2 | 0.1 | 0.16 |
|  | Total | 0.18 | 0.16 | 0.22 |
| R15 | LUL | 0.03 | 0.83 | 0.86 |
|  | LLL | 0.14 | 0.26 | 0.34 |
|  | RUL | 0.05 | 0.69 | 0.77 |
|  | RML | -0.04 | 0.76 | 0.82 |
|  | RLL | 0.12 | 0.34 | 0.42 |
|  | Total | 0.09 | 0.46 | 0.55 |
| R20 | LUL | -0.03 | 0.82 | 0.86 |
|  | LLL | 0.05 | 0.71 | 0.77 |
|  | RUL | -0.04 | 0.78 | 0.83 |
|  | RML | -0.12 | 0.36 | 0.45 |
|  | RLL | 0.02 | 0.87 | 0.88 |
|  | Total | 0 | 0.99 | 0.99 |
| X5 | LUL | -0.21 | 0.1 | 0.16 |
|  | LLL | -0.4 | <0.01 | <0.01 |
|  | RUL | -0.28 | 0.03 | 0.06 |
|  | RML | -0.22 | 0.08 | 0.14 |
|  | RLL | -0.37 | <0.01 | 0.01 |
|  | Total | -0.37 | <0.01 | 0.01 |
| X10 | LUL | -0.17 | 0.18 | 0.25 |
|  | LLL | -0.36 | <0.01 | 0.01 |
|  | RUL | -0.26 | 0.04 | 0.08 |
|  | RML | -0.18 | 0.15 | 0.22 |
|  | RLL | -0.35 | <0.01 | 0.01 |
|  | Total | -0.33 | <0.01 | 0.02 |
| X15 | LUL | -0.23 | 0.07 | 0.12 |
|  | LLL | -0.39 | <0.01 | <0.01 |
|  | RUL | -0.32 | 0.01 | 0.03 |
|  | RML | -0.24 | 0.06 | 0.11 |
|  | RLL | -0.41 | <0.001 | <0.01 |
|  | Total | -0.39 | <0.01 | <0.01 |
| X20 | LUL | -0.23 | 0.06 | 0.12 |
|  | LLL | -0.39 | <0.01 | <0.01 |
|  | RUL | -0.33 | <0.01 | 0.02 |
|  | RML | -0.24 | 0.05 | 0.11 |
|  | RLL | -0.43 | <0.001 | <0.01 |
|  | Total | -0.4 | <0.01 | <0.01 |
| f_res_ | LUL | 0.24 | 0.05 | 0.11 |
|  | LLL | 0.41 | <0.001 | <0.01 |
|  | RUL | 0.33 | <0.01 | 0.02 |
|  | RML | 0.24 | 0.06 | 0.11 |
|  | RLL | 0.41 | <0.001 | <0.01 |
|  | Total | 0.39 | <0.01 | <0.01 |
| A_x_ | LUL | 0.19 | 0.14 | 0.21 |
|  | LLL | 0.37 | <0.01 | <0.01 |
|  | RUL | 0.28 | 0.02 | 0.06 |
|  | RML | 0.2 | 0.12 | 0.18 |
|  | RLL | 0.38 | <0.01 | <0.01 |
|  | Total | 0.35 | <0.01 | 0.01 |
| Di5-20 | LUL | 0.13 | 0.31 | 0.4 |
|  | LLL | 0.32 | <0.01 | 0.03 |
|  | RUL | 0.23 | 0.07 | 0.12 |
|  | RML | 0.17 | 0.18 | 0.25 |
|  | RLL | 0.34 | <0.01 | 0.02 |
|  | Total | 0.29 | 0.02 | 0.05 |
| FVC% | LUL | -0.07 | 0.58 | 0.64 |
|  | LLL | -0.23 | 0.07 | 0.12 |
|  | RUL | -0.08 | 0.51 | 0.6 |
|  | RML | -0.08 | 0.55 | 0.64 |
|  | RLL | -0.19 | 0.13 | 0.19 |
|  | Total | -0.17 | 0.18 | 0.25 |
| FEV1% | LUL | -0.31 | 0.01 | 0.04 |
|  | LLL | -0.53 | <0.001 | <0.001 |
|  | RUL | -0.3 | 0.02 | 0.04 |
|  | RML | -0.29 | 0.02 | 0.05 |
|  | RLL | -0.42 | <0.001 | <0.01 |
|  | Total | -0.41 | <0.001 | <0.01 |
| FEV_1_/FVC | LUL | -0.54 | <0.001 | <0.001 |
|  | LLL | -0.68 | <0.001 | <0.001 |
|  | RUL | -0.59 | <0.001 | <0.001 |
|  | RML | -0.52 | <0.001 | <0.001 |
|  | RLL | -0.63 | <0.001 | <0.001 |
|  | Total | -0.65 | <0.001 | <0.001 |

Table S4. Multivariable regression analysis of associations between IOS variables and qCT-derived Jacobian.

|  |  | b | SE | t | p-value | R² |
| --- | --- | --- | --- | --- | --- | --- |
| R5 | LUL | -0.12 | 0.05 | -2.12 | 0.04 | 0.44 |
|  | LLL | -0.1 | 0.04 | -2.81 | <0.01 | 0.47 |
|  | RUL | -0.07 | 0.06 | -1.34 | 0.19 | 0.42 |
|  | RML | -0.15 | 0.06 | -2.61 | 0.01 | 0.46 |
|  | RLL | -0.11 | 0.04 | -2.87 | <0.01 | 0.48 |
|  | Total | -0.12 | 0.05 | -2.68 | <0.01 | 0.47 |
| R10 | LUL | -0.03 | 0.03 | -1.01 | 0.32 | 0.42 |
|  | LLL | -0.04 | 0.02 | -1.76 | 0.08 | 0.44 |
|  | RUL | -0.01 | 0.03 | -0.43 | 0.67 | 0.41 |
|  | RML | -0.04 | 0.03 | -1.35 | 0.18 | 0.42 |
|  | RLL | -0.04 | 0.02 | -1.87 | 0.07 | 0.44 |
|  | Total | -0.04 | 0.03 | -1.49 | 0.14 | 0.43 |
| R15 | LUL | -0.01 | 0.03 | -0.45 | 0.65 | 0.41 |
|  | LLL | -0.02 | 0.02 | -1.11 | 0.27 | 0.42 |
|  | RUL | 0 | 0.03 | -0.09 | 0.93 | 0.41 |
|  | RML | -0.02 | 0.03 | -0.84 | 0.4 | 0.42 |
|  | RLL | -0.02 | 0.02 | -1.22 | 0.23 | 0.42 |
|  | Total | -0.02 | 0.02 | -0.88 | 0.38 | 0.42 |
| R20 | LUL | 0 | 0.02 | 0.06 | 0.95 | 0.37 |
|  | LLL | -0.01 | 0.02 | -0.65 | 0.52 | 0.38 |
|  | RUL | 0.01 | 0.02 | 0.3 | 0.76 | 0.38 |
|  | RML | -0.01 | 0.03 | -0.37 | 0.71 | 0.38 |
|  | RLL | -0.01 | 0.02 | -0.73 | 0.47 | 0.38 |
|  | Total | -0.01 | 0.02 | -0.39 | 0.7 | 0.38 |
| X5 | LUL | 0.16 | 0.05 | 3.53 | <0.001 | 0.37 |
|  | LLL | 0.12 | 0.03 | 4.14 | <0.001 | 0.41 |
|  | RUL | 0.13 | 0.05 | 2.84 | <0.01 | 0.32 |
|  | RML | 0.19 | 0.05 | 4.07 | <0.001 | 0.4 |
|  | RLL | 0.13 | 0.03 | 4.07 | <0.001 | 0.4 |
|  | Total | 0.16 | 0.04 | 4.16 | <0.001 | 0.41 |
| X10 | LUL | 0.12 | 0.3 | 3.56 | <0.001 | 0.42 |
|  | LLL | 0.09 | 0.02 | 4.39 | <0.001 | 0.46 |
|  | RUL | 0.09 | 0.03 | 2.61 | 0.01 | 0.36 |
|  | RML | 0.13 | 0.03 | 4.02 | <0.001 | 0.44 |
|  | RLL | 0.1 | 0.02 | 4.34 | <0.001 | 0.46 |
|  | Total | 0.12 | 0.03 | 4.28 | <0.001 | 0.46 |
| X15 | LUL | 0.09 | 0.03 | 3.45 | <0.01 | 0.43 |
|  | LLL | 0.07 | 0.02 | 4.13 | <0.001 | 0.47 |
|  | RUL | 0.07 | 0.03 | 2.52 | 0.01 | 0.38 |
|  | RML | 0.1 | 0.03 | 3.73 | <0.001 | 0.44 |
|  | RLL | 0.07 | 0.02 | 4.13 | <0.001 | 0.47 |
|  | Total | 0.09 | 0.02 | 4.05 | <0.001 | 0.46 |
| X20 | LUL | 0.08 | 0.02 | 3.61 | <0.001 | 0.41 |
|  | LLL | 0.06 | 0.02 | 4.18 | <0.001 | 0.45 |
|  | RUL | 0.06 | 0.02 | 2.75 | <0.01 | 0.37 |
|  | RML | 0.09 | 0.02 | 3.72 | <0.001 | 0.42 |
|  | RLL | 0.06 | 0.02 | 4.16 | <0.001 | 0.45 |
|  | Total | 0.08 | 0.02 | 4.17 | <0.001 | 0.45 |
| f_res_ | LUL | -8.07 | 2.31 | -3.49 | <0.001 | 0.36 |
|  | LLL | -6.66 | 1.49 | -4.48 | <0.001 | 0.42 |
|  | RUL | -6.5 | 2.36 | -2.75 | <0.01 | 0.32 |
|  | RML | -9.12 | 2.38 | -3.83 | <0.001 | 0.38 |
|  | RLL | -6.78 | 1.55 | -4.38 | <0.001 | 0.42 |
|  | Total | -8.17 | 1.9 | -4.29 | <0.001 | 0.41 |
| A_x_ | LUL | -1.65 | 0.52 | -3.19 | <0.01 | 0.39 |
|  | LLL | -1.28 | 0.34 | -3.74 | <0.001 | 0.42 |
|  | RUL | -1.28 | 0.53 | -2.43 | 0.02 | 0.35 |
|  | RML | -1.96 | 0.53 | -3.69 | <0.001 | 0.42 |
|  | RLL | -1.33 | 0.35 | -3.77 | <0.001 | 0.42 |
|  | Total | -1.63 | 0.43 | -3.77 | <0.001 | 0.42 |
| Di5-20 | LUL | -0.12 | 0.04 | -2.97 | <0.01 | 0.42 |
|  | LLL | -0.09 | 0.03 | -3.43 | <0.01 | 0.45 |
|  | RUL | -0.08 | 0.04 | -2.01 | 0.05 | 0.38 |
|  | RML | -0.14 | 0.04 | -3.36 | <0.01 | 0.45 |
|  | RLL | -0.09 | 0.03 | -3.45 | <0.01 | 0.45 |
|  | Total | -0.11 | 0.03 | -3.44 | <0.01 | 0.45 |

Table S5. Multivariable regression analysis of associations between IOS variables and qCT-derived ADI.

|  |  | b | SE | t | p-value | R² |
| --- | --- | --- | --- | --- | --- | --- |
| R5 | LUL | -0.24 | 0.09 | -2.67 | <0.01 | 0.47 |
|  | LLL | -0.14 | 0.06 | -2.53 | 0.01 | 0.46 |
|  | RUL | -0.18 | 0.1 | -1.75 | 0.09 | 0.43 |
|  | RML | -0.22 | 0.07 | -3.16 | <0.01 | 0.49 |
|  | RLL | -0.19 | 0.06 | -3.03 | <0.01 | 0.48 |
|  | Total | -0.23 | 0.08 | -2.99 | <0.01 | 0.48 |
| R10 | LUL | -0.1 | 0.05 | -1.91 | 0.06 | 0.44 |
|  | LLL | -0.07 | 0.03 | -2.09 | 0.04 | 0.45 |
|  | RUL | -0.07 | 0.06 | -1.14 | 0.26 | 0.42 |
|  | RML | -0.08 | 0.04 | -1.98 | 0.05 | 0.44 |
|  | RLL | -0.08 | 0.04 | -2.37 | 0.02 | 0.46 |
|  | Total | -0.09 | 0.04 | -2.19 | 0.03 | 0.45 |
| R15 | LUL | -0.06 | 0.04 | -1.36 | 0.18 | 0.43 |
|  | LLL | -0.64 | 0.03 | -1.64 | 0.11 | 0.43 |
|  | RUL | -0.03 | 0.05 | -0.69 | 0.49 | 0.41 |
|  | RML | -0.05 | 0.04 | -1.42 | 0.16 | 0.43 |
|  | RLL | -0.06 | 0.03 | -1.88 | 0.06 | 0.44 |
|  | Total | -0.06 | 0.04 | -1.66 | 0.1 | 0.43 |
| R20 | LUL | -0.05 | 0.04 | -1.13 | 0.26 | 0.39 |
|  | LLL | -0.03 | 0.03 | -1.35 | 0.18 | 0.39 |
|  | RUL | -0.02 | 0.05 | -0.44 | 0.66 | 0.38 |
|  | RML | -0.04 | 0.03 | -1.13 | 0.26 | 0.39 |
|  | RLL | -0.04 | 0.03 | -1.49 | 0.14 | 0.4 |
|  | Total | -0.05 | 0.04 | -1.33 | 0.19 | 0.39 |
| X5 | LUL | 0.24 | 0.08 | 3 | <0.01 | 0.33 |
|  | LLL | 0.13 | 0.05 | 2.68 | <0.01 | 0.32 |
|  | RUL | 0.19 | 0.09 | 2.18 | 0.03 | 0.29 |
|  | RML | 0.23 | 0.06 | 3.88 | <0.001 | 0.39 |
|  | RLL | 0.18 | 0.05 | 3.43 | <0.01 | 0.36 |
|  | Total | 0.22 | 0.07 | 3.34 | <0.01 | 0.35 |
| X10 | LUL | 0.19 | 0.06 | 3.47 | <0.01 | 0.41 |
|  | LLL | 0.11 | 0.04 | 3.12 | <0.01 | 0.39 |
|  | RUL | 0.16 | 0.06 | 2.49 | 0.02 | 0.36 |
|  | RML | 0.18 | 0.04 | 4.22 | <0.001 | 0.45 |
|  | RLL | 0.14 | 0.04 | 3.79 | <0.001 | 0.43 |
|  | Total | 0.18 | 0.05 | 3.81 | <0.001 | 0.43 |
| X15 | LUL | 0.14 | 0.04 | 3.07 | <0.01 | 0.41 |
|  | LLL | 0.08 | 0.03 | 2.94 | <0.01 | 0.4 |
|  | RUL | 0.11 | 0.05 | 2.26 | 0.03 | 0.37 |
|  | RML | 0.13 | 0.03 | 3.68 | <0.001 | 0.44 |
|  | RLL | 0.11 | 0.03 | 3.58 | <0.001 | 0.44 |
|  | Total | 0.13 | 0.04 | 3.49 | <0.001 | 0.43 |
| X20 | LUL | 0.12 | 0.04 | 2.93 | <0.01 | 0.38 |
|  | LLL | 0.07 | 0.02 | 2.91 | <0.01 | 0.37 |
|  | RUL | 0.1 | 0.04 | 2.19 | 0.03 | 0.34 |
|  | RML | 0.11 | 0.03 | 3.51 | <0.001 | 0.41 |
|  | RLL | 0.1 | 0.03 | 3.58 | <0.001 | 0.41 |
|  | Total | 0.11 | 0.03 | 3.44 | <0.01 | 0.4 |
| f_res_ | LUL | -13.33 | 3.99 | -3.34 | <0.01 | 0.35 |
|  | LLL | -8.36 | 2.44 | -3.42 | <0.01 | 0.36 |
|  | RUL | -11.22 | 4.47 | -2.51 | 0.01 | 0.3 |
|  | RML | -12.1 | 3.06 | -3.96 | <0.001 | 0.39 |
|  | RLL | -10.75 | 2.63 | -4.08 | <0.001 | 0.4 |
|  | Total | -12.76 | 3.25 | -3.93 | <0.001 | 0.39 |
| A_x_ | LUL | -2.59 | 0.9 | -2.88 | <0.01 | 0.37 |
|  | LLL | -1.44 | 0.56 | -2.57 | 0.01 | 0.35 |
|  | RUL | -2.22 | 1 | -2.23 | 0.03 | 0.34 |
|  | RML | -2.51 | 0.69 | -3.66 | <0.001 | 0.41 |
|  | RLL | -1.94 | 0.61 | -3.19 | <0.01 | 0.39 |
|  | Total | -2.4 | 0.74 | -3.23 | <0.01 | 0.39 |
| Di5-20 | LUL | -0.2 | 0.07 | -2.89 | <0.01 | 0.42 |
|  | LLL | -0.11 | 0.04 | -2.59 | 0.01 | 0.41 |
|  | RUL | -0.16 | 0.08 | -2.11 | 0.04 | 0.39 |
|  | RML | -0.18 | 0.05 | -3.55 | <0.001 | 0.46 |
|  | RLL | -0.14 | 0.05 | -3.14 | <0.01 | 0.43 |
|  | Total | -0.18 | 0.06 | -3.19 | <0.01 | 0.44 |

Table S6. Multivariable regression analysis of associations between IOS variables and qCT-derived fSAD%.

|  |  | b | SE | t | p-value | R² |
| --- | --- | --- | --- | --- | --- | --- |
| R5 | LUL | 0 | 0.13 | -0.03 | 0.98 | 0.4 |
|  | LLL | 0.25 | 0.15 | 1.61 | 0.16 | 0.43 |
|  | RUL | 0.13 | 0.14 | 1.08 | 0.36 | 0.41 |
|  | RML | 0.12 | 0.12 | 0.98 | 0.41 | 0.41 |
|  | RLL | 0.31 | 0.16 | 1.96 | 0.08 | 0.44 |
|  | Total | 0.22 | 0.15 | 1.45 | 0.21 | 0.42 |
| R10 | LUL | -0.01 | 0.07 | -0.07 | 0.95 | 0.41 |
|  | LLL | 0.07 | 0.09 | 0.77 | 0.44 | 0.41 |
|  | RUL | 0 | 0.07 | 0.03 | 0.97 | 0.41 |
|  | RML | -0.01 | 0.07 | -0.16 | 0.87 | 0.41 |
|  | RLL | 0.08 | 0.09 | 0.87 | 0.39 | 0.41 |
|  | Total | 0.03 | 0.08 | 0.4 | 0.69 | 0.41 |
| R15 | LUL | -0.02 | 0.06 | -0.31 | 0.76 | 0.41 |
|  | LLL | 0.03 | 0.07 | 0.43 | 0.67 | 0.41 |
|  | RUL | -0.02 | 0.06 | -0.34 | 0.74 | 0.41 |
|  | RML | -0.03 | 0.06 | -0.54 | 0.59 | 0.41 |
|  | RLL | 0.03 | 0.08 | 0.36 | 0.72 | 0.41 |
|  | Total | 0 | 0.07 | -0.02 | 0.98 | 0.41 |
| R20 | LUL | -0.03 | 0.06 | -0.59 | 0.56 | 0.38 |
|  | LLL | 0 | 0.07 | -0.04 | 0.97 | 0.37 |
|  | RUL | -0.04 | 0.06 | -0.72 | 0.47 | 0.38 |
|  | RML | -0.05 | 0.05 | -0.89 | 0.38 | 0.38 |
|  | RLL | -0.02 | 0.07 | -0.29 | 0.78 | 0.38 |
|  | Total | -0.03 | 0.07 | -0.5 | 0.62 | 0.38 |
| X5 | LUL | -0.06 | 0.12 | -0.49 | 0.62 | 0.24 |
|  | LLL | -0.38 | 0.13 | -2.98 | <0.01 | 0.33 |
|  | RUL | -0.22 | 0.11 | -2.08 | 0.04 | 0.28 |
|  | RML | -0.25 | 0.11 | -2.38 | 0.02 | 0.3 |
|  | RLL | -0.42 | 0.13 | -3.15 | <0.01 | 0.34 |
|  | Total | -0.35 | 0.13 | -2.73 | <0.01 | 0.32 |
| X10 | LUL | -0.05 | 0.08 | -0.56 | 0.58 | 0.29 |
|  | LLL | -0.26 | 0.09 | -2.85 | <0.01 | 0.38 |
|  | RUL | -0.16 | 0.08 | -2.07 | 0.04 | 0.34 |
|  | RML | -0.16 | 0.08 | -2.03 | 0.05 | 0.34 |
|  | RLL | -0.31 | 0.1 | -3.19 | <0.01 | 0.39 |
|  | Total | -0.24 | 0.09 | -2.63 | 0.01 | 0.36 |
| X15 | LUL | -0.05 | 0.07 | -0.71 | 0.48 | 0.32 |
|  | LLL | -0.21 | 0.07 | -2.84 | <0.01 | 0.4 |
|  | RUL | -0.13 | 0.06 | -2.13 | 0.04 | 0.36 |
|  | RML | -0.12 | 0.06 | -1.93 | 0.06 | 0.35 |
|  | RLL | -0.25 | 0.07 | -3.38 | <0.01 | 0.42 |
|  | Total | -0.19 | 0.07 | -2.68 | <0.01 | 0.39 |
| X20 | LUL | -0.05 | 0.06 | -0.79 | 0.43 | 0.29 |
|  | LLL | -0.2 | 0.06 | -3.03 | <0.01 | 0.38 |
|  | RUL | -0.12 | 0.05 | -2.27 | 0.03 | 0.34 |
|  | RML | -0.1 | 0.05 | -1.84 | 0.07 | 0.32 |
|  | RLL | -0.23 | 0.07 | -3.53 | <0.001 | 0.41 |
|  | Total | -0.18 | 0.06 | -2.78 | <0.01 | 0.37 |
| f_res_ | LUL | 5.72 | 5.86 | 0.98 | 0.33 | 0.24 |
|  | LLL | 20.07 | 6.48 | 3.1 | <0.01 | 0.34 |
|  | RUL | 12.78 | 5.41 | 2.36 | 0.02 | 0.3 |
|  | RML | 9,89 | 5.45 | 1.82 | 0.07 | 0.27 |
|  | RLL | 23.05 | 6.69 | 3.45 | <0.01 | 0.36 |
|  | Total | 18.19 | 6.39 | 2.85 | <0.01 | 0.32 |
| A_x_ | LUL | 0.82 | 1.3 | 0.63 | 0.53 | 0.29 |
|  | LLL | 4.11 | 1.45 | 2.84 | <0.01 | 0.37 |
|  | RUL | 2.97 | 1.19 | 2.5 | 0.02 | 0.35 |
|  | RML | 2.78 | 1.18 | 2.35 | 0.02 | 0.34 |
|  | RLL | 5.15 | 1.47 | 3.5 | <0.001 | 0.41 |
|  | Total | 4.16 | 1.4 | 2.96 | <0.01 | 0.37 |
| Di5-20 | LUL | 0.03 | 0.1 | 0.32 | 0.75 | 0.34 |
|  | LLL | 0.25 | 0.11 | 2.21 | 0.03 | 0.39 |
|  | RUL | 0.17 | 0.09 | 1.91 | 0.06 | 0.38 |
|  | RML | 0.17 | 0.09 | 1.87 | 0.07 | 0.38 |
|  | RLL | 0.33 | 0.11 | 2.89 | <0.01 | 0.42 |
|  | Total | 0.25 | 0.11 | 2.28 | 0.03 | 0.39 |
